# Seasonality and the effects of weather on *Campylobacter* infections

**DOI:** 10.1186/s12879-019-3840-7

**Published:** 2019-03-13

**Authors:** Abdelmajid Djennad, Giovanni Lo Iacono, Christophe Sarran, Christopher Lane, Richard Elson, Christoph Höser, Iain R. Lake, Felipe J. Colón-González, Sari Kovats, Jan C. Semenza, Trevor C. Bailey, Anthony Kessel, Lora E. Fleming, Gordon L. Nichols

**Affiliations:** 1 Statistics, Modelling and Economics Department, National Infection Service, Public Health England, 61, Colindale Avenue, London, NW9 5EQ UK; 2 School of Veterinary Medicine, University of Surrey, Guildford, UK; 3 Met Office, Exeter, UK; 4 World Health Organisation, Geneva, Switzerland; 5 National Infection Service, Public Health England, London, UK; 6 NIHR Health Protection Research Unit in Gastrointestinal Infections, London, UK; 7 Institute for Hygiene and Public Health, GeoHealth Centre, University of Bonn, Bonn, Germany; 8 University of East Anglia, Norwich, UK; 9 London School of Hygiene and Tropical Medicine, London, UK; 10 European Centre for Disease Prevention and Control, Stockholm, Sweden; 11 University of Exeter, Exeter, UK; 12 University of Thessaly, Larissa, Thessaly Greece

**Keywords:** *Campylobacter*, Time series, Temperature, Rainfall, Climate change, Environmental health

## Abstract

**Background:**

*Campylobacteriosis* is a major public health concern. The weather factors that influence spatial and seasonal distributions are not fully understood.

**Methods:**

To investigate the impacts of temperature and rainfall on *Campylobacter* infections in England and Wales, cases of *Campylobacter* were linked to local temperature and rainfall at laboratory postcodes in the 30 days before the specimen date. Methods for investigation included a comparative conditional incidence, wavelet, clustering, and time series analyses.

**Results:**

The increase of *Campylobacter* infections in the late spring was significantly linked to temperature two weeks before, with an increase in conditional incidence of 0.175 cases per 100,000 per week for weeks 17 to 24; the relationship to temperature was not linear. Generalized structural time series model revealed that changes in temperature accounted for 33.3% of the expected cases of *Campylobacteriosis*, with an indication of the direction and relevant temperature range. Wavelet analysis showed a strong annual cycle with additional harmonics at four and six months. Cluster analysis showed three clusters of seasonality with geographic similarities representing metropolitan, rural, and other areas.

**Conclusions:**

The association of *Campylobacteriosis* with temperature is likely to be indirect. High-resolution spatial temporal linkage of weather parameters and cases is important in improving weather associations with infectious diseases. The primary driver of *Campylobacter* incidence remains to be determined; other avenues, such as insect contamination of chicken flocks through poor biosecurity should be explored.

**Electronic supplementary material:**

The online version of this article (10.1186/s12879-019-3840-7) contains supplementary material, which is available to authorized users.

## Background

The distinctive seasonal pattern of *Campylobacter* incidence has suggested that seasonal changes in the environment contribute to this pattern. The examination of the numbers of *Campylobacter* in the small intestine and caeca of chicken over a 12-month period showed significant seasonal variation in the carriage rate which was associated with sunshine and temperature [1]. The analysis of surveillance data from Europe, Canada, Australia and New Zealand found a lack of clear and consistent association with rainfall, humidity, sunshine, and temperature [2]. The analysis of *Campylobacter* data from Denmark, Sweden, Austria, Finland, New Zealand, Scotland, and Wales showed consistent seasonal patterns of infection with more cases in the summer, but with cases peaking at different times of the year [3]. *Campylobacter* rates in England and Wales were associated with temperature, sunshine, and rainfall, with temperature being the most significant variable and a 5% increase in *Campylobacter* cases was associated with a 1 °C rise in temperature to a maximum temperature of 14 °C [4–6].

An examination of the relationship between *Campylobacter* cases (along with *Salmonella* and pathogenic *Escherichia coli*) in Canada found an increase in cases of 2.2% for every degree of increase in temperature, with a low temperature threshold of 10 °C; another Canadian study modelled *Campylobacter* and temperature and found an association with temperature above a threshold of 10 °C [7, 8]. *Campylobacter* incidence in Massachusetts closely followed the peak in annual temperature with a lag of 2–14 days, and in Georgia (US) a strong association was found between temperature and *Campylobacter* infections [9, 10].

The impact of weather parameters on *Campylobacter* differed between temperate and sub-tropical areas of Australia [11]. A spatial examination of *Campylobacter* in New Zealand found no association with climate variables. Temperature did not appear to drive the seasonality, and there was little association with rainfall [12–14]. Jore et al. [15] showed a relationship between temperature with both human infections and chicken colonisation in six European countries. There has also been work in Switzerland suggesting the seasonality of *Campylobacter* is partly explained by contamination of chicken broilers [16].

While an association with weather in general, and temperature in particular, appears to be a relatively common finding in these studies, nevertheless the link is not as clear, consistent and direct as with Salmonella; nor does it have a well-defined mechanism. Nichols [17] and Ekdahl et al. [18] suggested that flies might explain the epidemiology of *Campylobacter*, through the direct transfer of contaminated material from raw meats or faeces to ready-to-eat foods, and/or by the seasonal contamination of chicken flocks [19–21]. Nichols [17] proposed a fly transmission model that explained the seasonal distribution by the impact of temperature on non-biting fly numbers, as measured by the development time of *Musca domestica* larvae. Evidence suggested that the use of fly screens on chicken production houses could reduce the seasonal contamination of chickens and subsequent human disease significantly [21]. Similar responses to temperature in both chicken broiler carcase contaminations with *Campylobacter* and human infections with *Campylobacter* cases in six different European Countries, implying that the seasonal relationship between human infection and temperature is more likely a result of exposure to chicken [15].

One of the additional complicating factors in the *Campylobacter* transmission in most developed countries is the significant percentage of cases related to foreign travel, and the seasonality of this additional factor [22, 23]. Hartnack et al. [24] highlighted the necessity of quantifying the transmission of *Campylobacter* from broiler to humans and to include climatic factors in order to gain further insight in its epidemiology. Sterk et al. [25] used a runoff simulator to analyse the effect of climate change on *Campylobacter* (along with *Cryptosporidium*) runoff and human infection risks in the Netherlands; and found that climate change has little overall impact on runoff of *Campylobacter* (along with *Cryptosporidium*) from land to the surface waters.

Within climate change research, there are three reasons for undertaking this work. Firstly to demonstrate evidence of the potential short-term consequences on human health from climate change; secondly to understand what factors influence the occurrence of infectious diseases; and thirdly to provide models which can forecast future disease risks. The incidence of *Campylobacter* in the UK demonstrated both a strong and regular seasonality, and a variation in the number of cases per year [4, 6, 26]. This raises a question of whether the seasonal patterns of *Campylobacter* have changed over time as a result of climate change and other environmental factors.

## Methods

*Campylobacter* surveillance data were extracted from the Second Generation Surveillance System (SGSS) database of Public Health England from 1989 to 2014. A case was defined as a human faecal sample submitted from a patient that was positive for *Campylobacter*. All *Campylobacter* surveillance data were laboratory-confirmed cases and were predominantly based on the isolation of *Campylobacter* from faeces using selective culture media in a micro aerobic environment; membrane filter, PCR or other approaches made up a very small percentage of cases. A few sentinel laboratory sites conducted screening using a range of PCR targets during the 2012 Olympic Games. Standard antimicrobial testing methods, used in primary diagnostic laboratories, were predominantly disc diffusion methods. Where speciation was reported, it was conducted in diagnostic laboratories using conventional phenotypic methods [6]. Most cases were thought to be symptomatic and included patients with extra intestinal infections [6]. The symptoms were those recorded on the physician request form and generally are in the simplistic form of “D & V” or something equivalent.

The meteorological data were supplied by the Met Office and held on the Medical and Environmental Data a Mash-up Infrastructure (MEDMI) platform (see https://www.data-mashup.org.uk).

Reported cases between 2005 and 2009 were linked to local weather parameters at the laboratory postcode locations; namely: maximum, minimum and average daily temperature and daily rainfall in 30 days before the specimen date using a previously published approach that prevented patient identification [6]. The linkage was conducted on laboratory postcode because this was more complete than patient residential postcode and preserved patient confidentiality. An additional study [27] validated that the weather parameters at the laboratory postcode correlate well with the weather at the domestic postcode of cases.

### Comparative conditional incidence

GIS analysis was performed on cases with Lower Layer Super Output Areas (LSOA) using ArcGIS 10.2 for Desktop by ESRI. A map of *Campylobacter* incidence between 2005 and 2009 from patient’s residential postcode data was produced. Some areas had poor residential postcode completeness resulting in areas with an apparent lower incidence. Comparative conditional incidence **(**CCI) is a new approach which uses cases and LSOA populations to examine differences between disease incidence at different temperature and rainfall in the 30 days before the specimen date within equal sized datasets. CCI is a sum of cases per week within all LSOAs with same temperature divided by sum of population in the same LSOAs. It is conditional as the weather variables were available for the weeks and LSOAs where there were positive cases and excluded patients without a residential postcode as it was not possible to establish the LSOA.

### Wavelet analysis

To detect potential time-varying seasonality in *Campylobacter* that might result from *Salmonella* control measures in chicken production, such as improved biosecurity practices, we used wavelet analysis. *Campylobacter* daily counts between 1989 and 2009 were adjusted from the day of the year using a seven-day moving average and systematic adjustments for the reduced reporting over bank holidays and for long-term trends as previously reported [6]. The analyses were conducted in R using ‘waveletComp’ [28].

### Generalized structural time series model

*Campylobacter* cases across ten geographical regions in England and Wales were examined using the generalized structural time series model (GEST) [29] with a negative binomial distribution, where the natural logarithm of the expected cases of *Campylobacter* was decomposed into baseline, trend and seasonal components. To investigate the effect of weather variables on the development of *Campylobacter* cases, explanatory variables of the average temperature (°C) and the total rainfall (mm), of one, two, three and four weeks before diagnosis were included in the model. The temperature variable was examined using fixed and varying-coefficient analyses in order to account for variations in the relationship through the year.

### Cluster analysis

We examined the geographic similarity of *Campylobacter* seasonality in England and Wales in thirty-three sub-regional areas between 1989 and 2009. The fitted seasonality of *Campylobacter* infections across thirty-three Strategic Health Authorities were plotted in a dendrogram using hierarchical clustering analysis with Ward’s minimum variance. Clusters were drawn up that exhibited a distance of one or more from other clusters; and this was used to examine the seasonality of groups of SHAs. Additional file 1: Section S4 illustrates an example of the GEST analysis of *Campylobacter* cases in Avon, Gloucestershire and Wiltshire SHA. The clustering was conducted using the ‘hclust’ routine of the stats package in R statistical software. Table 1 illustrates methods used, time period, geography, and reason for choice in the analysis.

**Table 1:** Methods, time, geography, and reason for choice in the analyses

Methods	Time	Spatial unit	Reason for choice
GIS incidence mapping	2005–2014	Lower Layer Super Output Areas (LSOAs)	Individual resident postcodes (required for mapping by population) were not available before 2005.
Local linkage of cases and weather	2005–2009	Laboratory postcode	Local linkage of cases and weather variables through the laboratory postcode was available from 2005 to 2009 in ten geographical regions.
Comparative conditional incidence	2005–2009	LSOAs	Lack of negative cases, positive cases were linked to weather variables from 2005 to 2009.
GEST model	2005–2009	Laboratory postcode	Local linkage of cases and weather data through the laboratory postcode was available from 2005 to 2009 in ten geographical regions.
Wavelet analysis	1989–2009	England and Wales	The cases were not linked to weather variables. Health and weather data were analysed separately.
Ward’s minimum variance clustering & GEST	1989–2009	Strategic Health Authorities postcode	To increase the number of regions from ten to thirty-three sub-regions and determine the clustering of geographic similarities of the seasonality. Cases were not linked to weather variables.

## Results

**Fig. 1:**
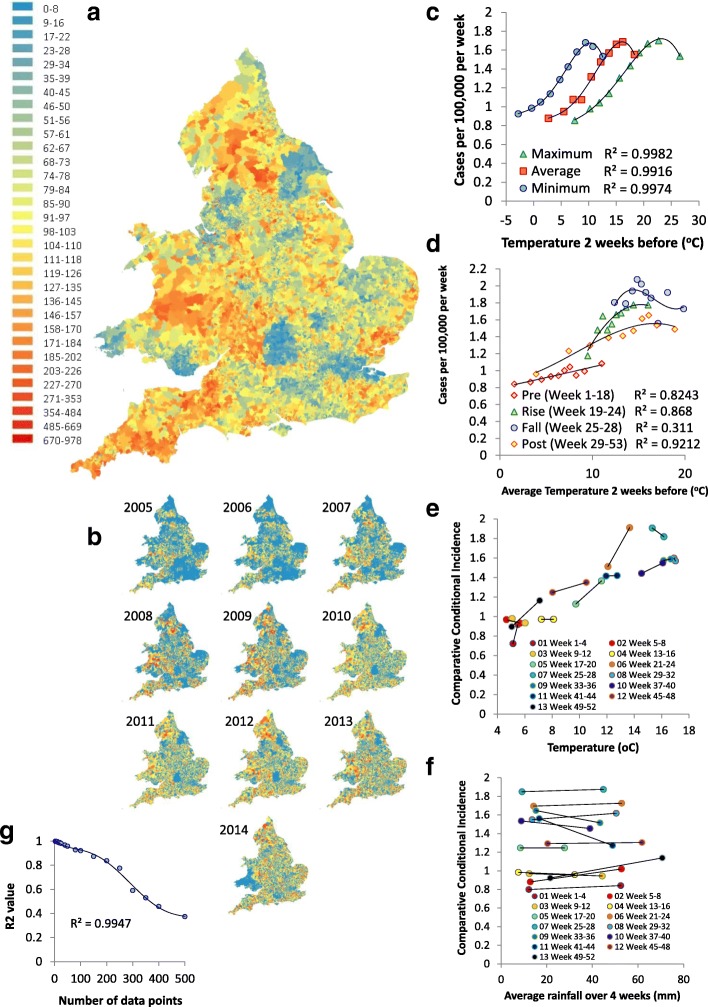
Incidence. **a** Average *Campylobacter* cases per 100,000 population for the 10 years 2005 to 2014 by Lower Layer Super Output Area (LSOA) in England and Wales; **b** *Campylobacter* cases per 100,000 per year from 2005 to 2014; **c** Comparative conditional incidence (CCI) of *Campylobacter* distributed evenly over ten data points and sorted by maximum, average and minimum temperatures two weeks before. **d** CCI of *Campylobacter* on four separate periods plotted against average temperature two weeks before; **e** CCI of *Campylobacter* on four-week periods splitting each dataset into two equal parts of temperature two weeks before. **f** CCI *Campylobacter* on four-week periods splitting each dataset into two equal parts of average rainfall four weeks before; **g** R2 values using different numbers of data points based on the average temperature two weeks before

Incidence was determined from patient residence postcode data (2005–2014) and plotted by LSOA across England and Wales. The geographic representation of cases per 100,000 per week showed areas of the country with an apparently low incidence (Fig. 1a; shown in blue); this is presumed to be where residential postcode data were missing and/or there were under-reporting. Data on individual years of cases showed a gradual improvement in the completeness of reporting from 2005 to 2014 (Fig. 1b).

The CCI was estimated using datasets that split cases into ten equal parts by temperature and rainfall. The highest CCI values were found for the minimum temperature (9 °C) two weeks before the specimen date, average temperature (16 °C) two weeks before the specimen date and maximum temperature (21 °C) two weeks before the specimen date (Fig. 1c). The cases at the height of summer when temperatures were highest had lower cases than a few weeks earlier. Between average temperatures of 5 °C and 15 °C, there was a 0.09 case increase in the incidence per 100,000 per week (Fig. 1d). It is unclear how this relates to the actual incidence as the LSOA per weeks that did not have a *Campylobacter* case which could not be included. Using four week periods, average temperatures two weeks before and splitting the data for each time period into two equal halves by temperature, demonstrated that the relationship between temperature and incidence in each time period differed, forming a cyclic loop (Fig. 1e). For weeks 17 to 20 and 21 to 24 there was an increase in the CCI of 0.175 cases per 100,000 per week for each degree increase in the temperature two weeks before (Fig. 1e). A similar approach using average rainfall over the previous four weeks showed no consistent links with the CCI (Fig. 1f). Adjusting the number of data points to give larger or smaller percentages of the data in each point affected the R^2^ value of the CCI on all data, with optimum results for 200 data points or less (Fig. 1g).

**Fig. 2:**
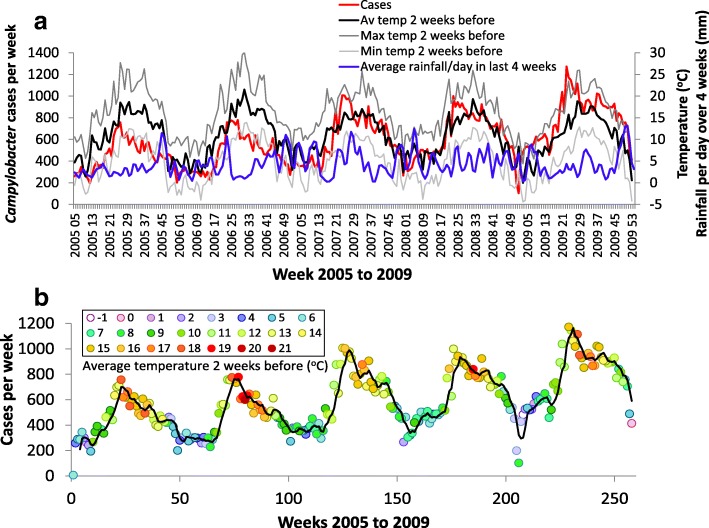
*Campylobacter* cases and weather. **a** *Campylobacter* cases per week from 2005 to 2009 in England and Wales; average rainfall per day in the previous four weeks; maximum, minimum and average temperature two weeks before the specimen date; **b** Cases per week and average temperature two weeks before the specimen date associated with cases

The number of *Campylobacter* cases per week from 2005 to 2009 in England and Wales, average rainfall (mm) in the previous four weeks, maximum, minimum and average temperature (°C) in the two weeks before the specimen date are shown in Fig. 2a. Numbers of cases per week and average temperature two weeks previously associated with cases on colour are shown in Fig. 2b.

**Fig. 3:**
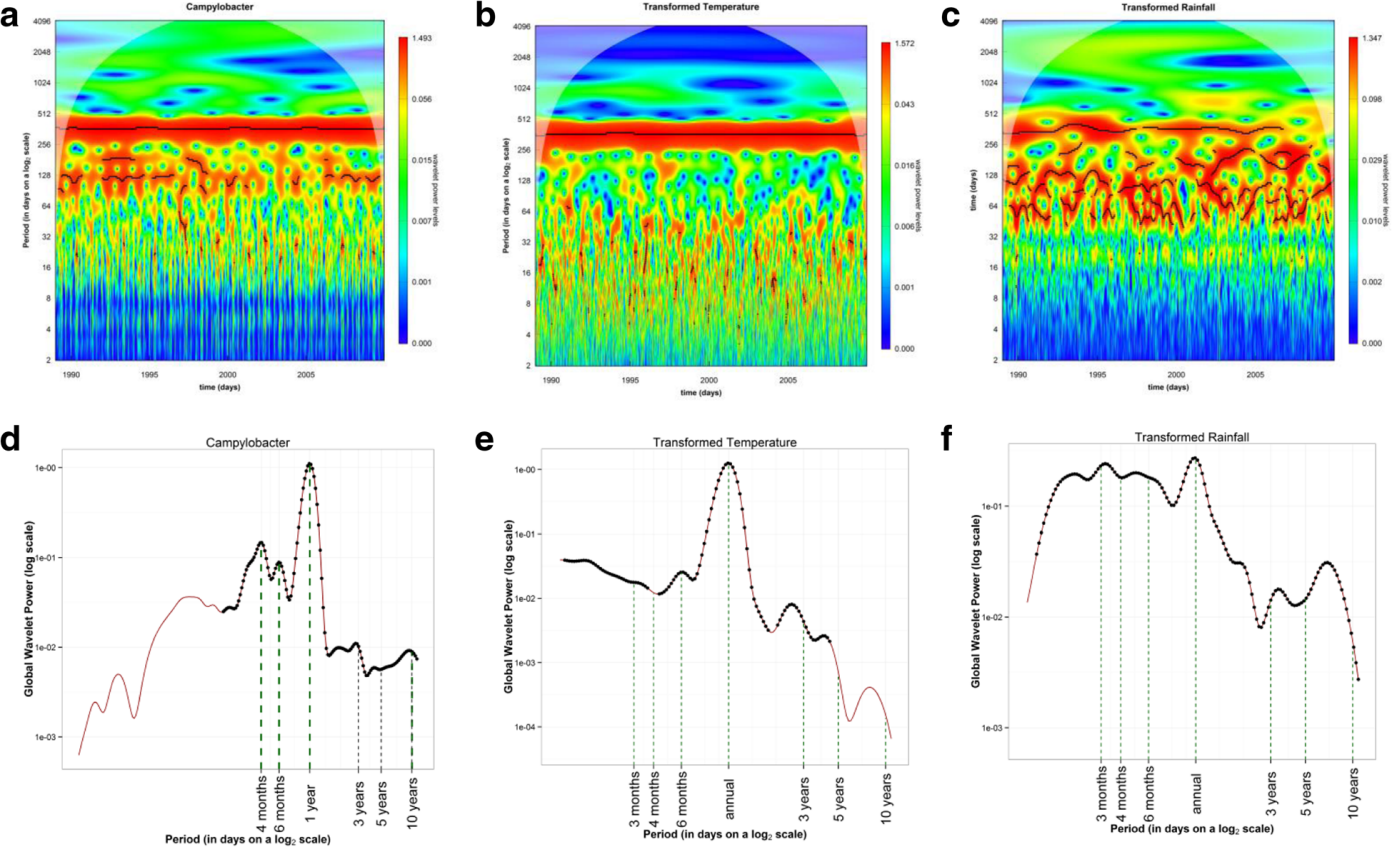
Wavelet analysis for *Campylobacter* cases reported between 1989 and 2009 and for temperature and rainfall in England and Wales; **a** Wavelet power spectrum of the root transformed time-series of daily *Campylobacter* cases adjusted using a seven-day rolling mean, removal of bank holiday artefacts and adjusted for long term trend; **b** Wavelet power spectrum of the root transformed time-series of daily temperature; **c** of daily Rainfall. Low values of the power spectrum are shown in dark blue and high values in dark red. The black lines show the maxima of the undulations of the wavelet power spectrum. The light white shaded areas identify the region influenced by edge; **d** Global average wavelet power spectrum of the root transformed time-series of *Campylobacter* cases, the black dots show the 5% significant levels computed based on 500 bootstrapped series; **e** as **d** but for averaged temperature

Wavelet analysis of *Campylobacter* cases showed a strong annual periodicity with smaller semi-annual and 4 monthly periodicities, i.e. the ‘harmonics’ corresponding to the red horizontal regions in the power spectrum (Fig. 3a) and the three main peaks in the global power spectrum (Fig. 3d) located at 365, 180 and 121 days. Furthermore, wavelet analysis for temperature showed a strong annual seasonality but was also dominated by semi-annual. However, there were years (e.g. from April 2004 to July 2008) where this component was absent in the temperature, but present in the *Campylobacter* cases. Rainfall data over the same time period exhibited different patterns, except for the annual seasonality.

GEST with single coefficients for temperature and rainfall found a statistically significant association between *Campylobacter* and temperature of two weeks before the case diagnosis (see Additional file 1: Table S1). The relationship between *Campylobacter* and temperature was positive, as for every degree increase in average weekly temperature (°C) of two weeks before the specimen date there is an increase of 0.73% in the mean number of *Campylobacter* cases. For rainfall, there was a slightly significant association between *Campylobacter* and total rainfall of one week before the case diagnosis. The relationship was negative, as for every millimetre increase in total rainfall of one week before the specimen date there is a decrease of 0.24% in the mean number of *Campylobacter* cases.

Additional file 1: Table S2 provides clear evidence for an association between *Campylobacter* and average, maximum and minimum temperatures of two weeks before the case diagnosis in most geographical regions of England. Wales did not show significant association. All the regions showed statistically significant increasing trend in cases except the East of England. Additional file 1: Figure S1 shows the weekly cases of *Campylobacter* in England and Wales with the fitted mean in red (panel a), and the decomposition of the fitted mean into fitted trend (panel e), fitted seasonality (panel f), fitted linear effect of average temperature (panel c) and fitted linear effect of total rainfall (panel d). Expected cases of *Campylobacter* increased with the average temperature two weeks before diagnosis above a threshold of 11 °C. The relative change in the expected cases of *Campylobacter* due to temperature and rainfall in England and Wales were 7.7% and − 1.9% respectively and the effect of seasonality was − 3.4% (see Additional file 1: Table S3).

**Fig. 4:**
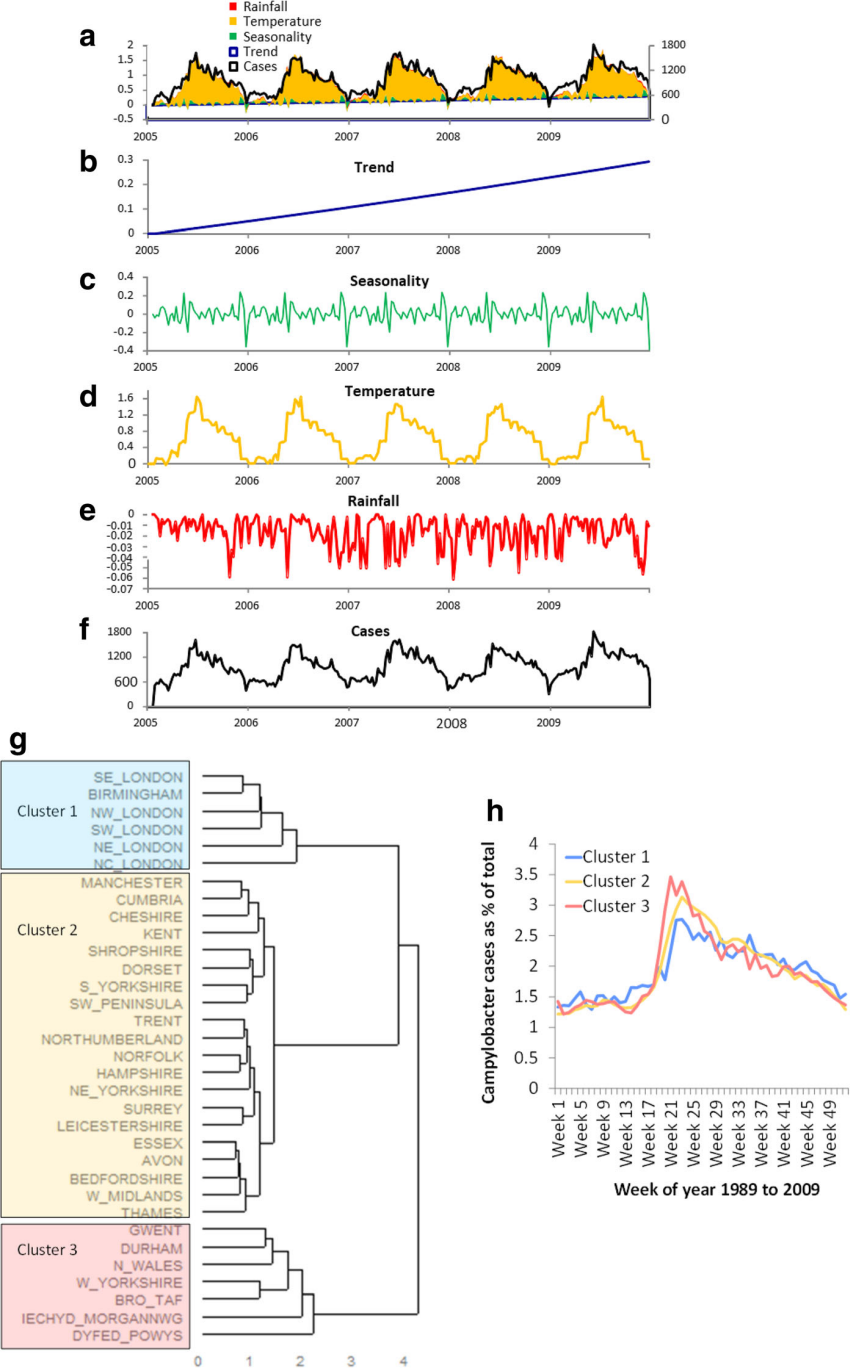
GEST analysis for *Campylobacter* cases in England and Wales from 2005 to 2009 using temperature and rainfall as explanatory variables, and seasonal hierarchical clustering. **a** Cases with the fitted mean decomposed by trend, seasonality, temperature and rainfall; **b** Fitted linear trend in time; **c** Fitted seasonality; **d** Fitted varying coefficients for temperature; **e** Fitted fixed coefficient for rainfall; **f** *Campylobacter* cases; **g** Sub-regional clustering by seasonality using Ward’s Minimum Variance based on the GEST model. **h** Seasonal distribution of *Campylobacter* cases in the three main clusters

In GEST with varying coefficients of temperature, using 13 four week periods as factors through the year and a single coefficient for rainfall, the relative change in the expected cases of *Campylobacter* due to temperature in England and Wales increased to 33.3%, the effect of rainfall was − 1.7%, and the effect of seasonality was − 0.5% (see Additional file 1: Table S4). Figure 4 shows the weekly cases of *Campylobacter* in England and Wales and the fitted mean with varying coefficients of temperature, rainfall, seasonality and trend, and the decomposition of the fitted mean. The fitted effect of 13 four week periods on temperature is shown in Additional file 1: Figure S2c.

The Ward’s minimum variance clustering of the fitted seasonality of the GEST showed three main clusters: metropolitan (London and Birmingham), rural (Wales and the North East), and other geographical areas (Fig. 4g). When cases were plotted by week of year for the three clusters and the same time period, the seasonal distribution differed significantly (Fig. 4h).

## Discussion

The epidemiology of *Campylobacter* in humans remains a subject of importance because it is the commonest bacterial cause of diarrhoea in most developed countries, and because intervention requires an understanding of disease transmission pathways and possible association with climate and other environmental change. Within the context of climate change research, the approaches to linking weather parameters with infectious disease data commonly rely on an assumption that the relationships are continuous throughout the year, sometimes assuming a threshold value above which the continuous relationship changes. Here, we used different methods to demonstrate that *Campylobacter* incidence changes dynamically through the season, and we were able to decouple the relationship of temperature from the seasonality. Most notable was the very steep increase in the number of cases diagnosed between weeks 19 and 24, although there was still a relationship to temperature before this (weeks 1-18) as well as later in the year (weeks 29-53). The distribution was also consistent with the direct and/or indirect fly transmission hypotheses [17–19], with the fly populations increasing during the late spring period resulting in transmission passing from faeces or other contaminated material to either food and/or chicken flocks (as a result of poor biosecurity). The expression of weekly incidence by the temperature during that week in an LSOA and averaged for all weeks with one or more *Campylobacter* cases, gives a means of examining the relationship between case numbers and temperature that is partially decoupled from seasonality and geography.

The GEST provides a useful approach for modelling over-dispersed counts with a negative binomial distribution. It allows for the extraction of hidden trend and seasonality and estimating the effect of weather parameters. The use of multilevel factors for temperature separated into 13 four week periods allowed the contribution of temperature two weeks prior to the diagnosis to be estimated, explaining that 33.3% of the contribution to the annual burden of *Campylobacter* (calculated as relative change in the predictive mean) was attributable to temperature, compared to 7.7% using a single coefficient value. The remaining regular seasonality across the five years was small, and was limited to holiday periods, perhaps reflecting surveillance bias. The contribution of rainfall to the incidence of *Campylobacter* was small. While being able to predict cases from the temperature in a much more complete way than the GEST model with a fixed coefficient, the GEST with the varying coefficient model did not provide an explanation of the causes of these changes, and suggested that other drivers linked to temperature were important.

When the GEST model was applied to weekly number of cases of *Campylobacter* from 1989 to 2009 in thirty three Strategic Health Authorities, it allowed the fitted seasonality to be used to group sub-regional SHAs into three geographical clusters.

The distribution of cases across geographically distinct sub-regions demonstrated strong differences in the strength of the seasonal pattern, but not the week when cases increase. The seasonality was strongest in Wales and the North East, and weakest and a bit more delayed in the large urban areas of London and Birmingham, with other geographic areas distributed in between. This implies that there are local factors that also influence *Campylobacter* disease occurrence. These could be demographic (e.g. more young people in cities), different risk profiles (e.g. more exposure to manures in the countryside; greater foreign travel in cities), and/or different impacts of weather locally (e.g. fly populations causing direct transmission to food or indirect transmission through the contamination of chicken flocks especially in rural areas) [17, 18, 21, 30].

Wavelet analysis showed that *Campylobacter* cases could be interpreted as the super-imposition of three main harmonics of annual, semi-annual and 4-months periods. The origin of these harmonics is not clear; however, annual and higher order (1/2 year, 1/3 year, etc.) harmonics, whose relative importance depends on the geographic region, are typical features of a range of climatic variables. Potential other drivers could be other environmental variables, reporting bias, human behaviour, and/or intrinsic seasonal patterns in animal/vector reservoirs (i.e. the contamination of chickens needs to be investigated). In this type of study the quality of data is important. We used laboratory postcode as a surrogate for patient residence postcode to anonymise the data and improve data completeness in linking cases to weather parameters in 30 days before the specimen date. Djennad et al. [27] demonstrated that it is valid to use laboratory postcode as a proxy for patient postcode. The algorithm to locally link case and weather was limited to a five-year period, for three temperature parameters and one rainfall parameter and had a lag of 30 days. Another limitation is that the data linkage was in cases only, with the result that weeks without cases were not recorded.

Being able to link cases to an LSOA allows the CCI to be applied to compare incidence using a range of weather parameters. This method utilised the geographic variation in weather parameters by combining data from small areas and their populations that had the same weather conditions. The CCI is limited by the weather data only being linked to cases only and not to the underlying population. While the approach provides a useful way of examining weather variables that are de-coupled from seasonality by grouping cases per week and their underlying populations at a particular temperature or rainfall level together as a combined incidence, it is clear that some weeks and areas will be left out because there are no *Campylobacter* cases. It would be expected that a measurement of the true incidence would give a result that differed somewhat from the CCI because the true incidence would include all the weeks with negative results not just those with positive ones. An extended version of this that uses weather parameters for both cases and populations has the potential to provide a more robust representation of the incidence of *Campylobacter* at different temperatures through the year.

The CCI and GEST approaches gave good results in examining environmental drivers for *Campylobacter*. Adding other weather parameters to these models should further improve the predictive power of these models or methods. As an example, the development of standard weather database and algorithms through the MEDMI project (https://www.data-mashup.org.uk) facilitates data linkage of environment and health data such that different analyses may be examined in a more systematic way for a wider range of infectious diseases [31]. The PHE *Campylobacter* dataset is sufficiently large, nevertheless the epidemiology of *Campylobacter* remains enigmatic and interventions have had limited success to date. It is therefore an important organism to study to elucidate methodologies that have power and utility in explaining infectious disease and climate change epidemiology for other infectious diseases in the future.

## Conclusions

The study provides strong association between *Campylobacter* and temperature. Using a range of statistical methods, the study suggests that temperature and/or rainfall alone cannot explain the entire seasonal variation of *Campylobacteriosis* risk in England and Wales. Further research should investigate if the temporal dependency of the relationship between *Campylobacter* incidence and temperature on the week might be driven by other environmental variables, or perhaps by an intrinsic seasonality in the dynamics of the bacterial population in the environment or in the zoonotic reservoir or potential vectors such as flies.

## Additional files

Additional file 1: **Figure S5** shows: a) Average weekly reported *Campylobacter* cases averaged over 20 years (from 1989 to 2009). All time series were square root transformed and then normalised to sum to unity. c) wavelet power spectrum of the transformed time-series of *Campylobacter*. Low values of the power spectrum are shown in dark blue, and high values in dark red. The black lines show the maxima of the undulations of the wavelet power spectrum. The light white shaded areas identify the region subjected to errors arising from dealing with a finite-length time series (edge effect). e) global average wavelet power spectrum, the black dots show the 5% significant levels computed based on 100 bootstrapped series g) original and reconstructed time-series according to all harmonics and the selected first 3 harmonics only. b), d), f) h) As in figures a), c) e) and g) but after the time-series of *Campylobacter* cases were adjusted using a seven day rolling mean, removal of bank holiday artefacts and adjusted for long term trend. (PDF 1032 kb)

## Abbreviations

CCI: Comparative Conditional Incidence
GEST: Generalized structural time series model
GIS: Geographic Information System
LSOA: Lower Layer Super Output Areas
PHE: Public Health England
SHA: Strategic Health Authorities
WMVC: Ward’s Minimum Variance Clustering
