# Supplementary material for: Seasonality and the effects of weather on Campylobacter infections
Source: BMC Infect Dis. 2019 Mar 13;19:255. doi: 10.1186/s12879-019-3840-7 (PMC6417031; doi:10.1186/s12879-019-3840-7)
Supplement: Supplementary file 1 — Figure S5 shows: a) Average weekly reported Campylobacter cases averaged over 20 years (from 1989 to 2009). All time series were square root transformed and then normalised to sum to unity. c) wavelet power spectrum of the transformed time-series of Campylobacter. Low values of the power spectrum are shown in dark blue, and high values in dark red. The black lines show the maxima of the undulations of the wavelet power spectrum. The light white shaded areas identify the region subjected to errors arising from dealing with a finite-length time series (edge effect). e) global average wavelet power spectrum, the black dots show the 5% significant levels computed based on 100 bootstrapped series g) original and reconstructed time-series according to all harmonics and the selected first 3 harmonics only. b), d), f) h) As in figures a), c) e) and g) but after the time-series of Campylobacter cases were adjusted using a seven day rolling mean, removal of bank holiday artefacts and adjusted for long term trend. (PDF 1032 kb) [file 12879_2019_3840_MOESM1_ESM.pdf]

Supplementary Material
Seasonality and the effects of weather on *Campylobacter* infections

Abdelmajid Djennad; Giovanni Lo Iacono; Christophe Sarran; Christopher Lane; Richard Elson; Christoph Höser; Iain Lake; Felipe J Colón-González; Sari Kovats; Jan C Semenza; Trevor C Bailey; Anthony Kessel; Lora E Fleming; Gordon L Nichols

Contents

S1 GEST model **2**

S2 Application of GEST to *Campylobacter* infections **2**
S2.1 GEST with fixed coefficient of temperature and rainfall 3
S2.2 GEST with varying coefficient of temperature 6

S3 The contribution of temperature, rainfall, trend and seasonal terms to the burden of *Campylobacter* **8**

S4 Hierarchical clustering analysis **10**
S4.1 Avon, Gloucestershire and Wiltshire SHA 10

S5 Wavelet analysis **12**

S1 GEST model

The generalized structural time series (GEST) model assumes that, conditional on the past, the response variable Y_t comes from a parametric distribution with probability (density) function $f_{Y_t}(y_t|\boldsymbol{\theta_t})$, where $\boldsymbol{\theta_t}$ is a vector of unknown distribution parameters. Here $\boldsymbol{\theta_t}$ is restricted to two parameters: $\boldsymbol{\theta_t} = (\mu_t, \sigma_t)$, where μ_t is in general a location parameter, σ_t a scale parameter. Each parameter (μ_t, σ_t) is modelled by a structural time series model and/or linear, non-linear or smooth non-parametric models to account for explanatory variables. Each structural model is a random walk or autoregressive model, and a random seasonality.

Definition: *Let Y_t be the dependent response variable for $t = 1, 2, \ldots, T$ then the GEST model of two parameters (μ_t, σ_t), defined as:*

$$
\begin{aligned}
Y_t|\mu_t, \sigma_t &\sim \mathcal{D}(\mu_t, \sigma_t) \\
g_1(\mu_t) &= \mathbf{x}_{1,t}^{\top}\boldsymbol{\beta}_1 + \gamma_{1,t} + s_{1,t} \\
\gamma_{1,t} &= \sum_{j=1}^{J_1} \phi_{1,j}\gamma_{1,t-j} + b_{1,t} \\
s_{1,t} &= -\sum_{m=1}^{M-1} s_{1,t-m} + w_{1,t} \\
g_2(\sigma_t) &= \mathbf{x}_{2,t}^{\top}\boldsymbol{\beta}_2 + \gamma_{2,t} + s_{2,t} \\
\gamma_{2,t} &= \sum_{j=1}^{J_1} \phi_{2,j}\gamma_{2,t-j} + b_{2,t} \\
s_{2,t} &= -\sum_{m=1}^{M-1} s_{2,t-m} + w_{2,t} \qquad \text{(S1)}
\end{aligned}
$$

where $\mathcal{D}$ represents the conditional distribution of the response variable, for $k = 1, 2$, g_k is a known link function (e.g., identity or log link function), $\boldsymbol{\beta}_k$ is a parameter vector of length p_k, $\mathbf{x_{k,t}}$ are explanatory variable vectors, $\gamma_{k,t}$ is the unobserved autoregressive (or random walk) trend of order J_k, $s_{k,t}$ is the time-varying seasonality, $b_{k,t}$ and $w_{k,t}$ are independently distributed disturbance terms with mean zero and variances $\sigma^2_{b_k}, \sigma^2_{w_k}$ where $\mathbf{b}_k \sim N_{T-J_k}\left(0, \sigma^2_{b_k}\mathbf{I}_{T-J_k}\right)$, $\mathbf{w}_k \sim N_{T-J_k}\left(0, \sigma^2_{w_k}\mathbf{I}_{T-J_k}\right)$, $\mathbf{I}_{T-J_k}$ is the unit matrix of size $T - J_k$.

S2 Application of GEST to *Campylobacter* infections

Here Y_t is the weekly number of cases of Campylobacter infections in England and Wales from February 2005 to December 2009, $\mathcal{D} = \mathcal{NBI}(\mu_t, \sigma_t)$ is a negative binomial type I distribution, the explanatory variables are maximum, minimum,

and average temperature and the total rainfall of, one, two, three and four weeks before diagnosis, the dispersion parameter is assumed to be constant.

S2.1 GEST with fixed coefficient of temperature and rainfall

$$
\begin{aligned}
Y_t|\mu_t, \sigma_t &\sim \mathcal{NBI}(\mu_t, \sigma_t) \\
\log(\mu_t) &= \beta_{1,0} + \beta_{1,1}(temperature_{t-h}) + \beta_{1,2}(rainfall_{t-l}) + \beta_{1,3}(\zeta_t) + s_{1,t} \\
s_{1,t} &= -\sum_{m=1}^{M-1} s_{1,t-m} + w_{1,t} \\
\log(\sigma_t) &= \beta_{2,0}
\end{aligned} \tag{S2}
$$

where $\zeta_t = 1, 2, \ldots, T$ is a fixed linear trend of time, $s_{1,t}$ is the random seasonality with frequency $M = 52$ and a random error term $w_{1,t} \sim N_T\left(0, \sigma_w^2 \mathbf{I}_T\right)$, h and l are the number of weeks before diagnosis.

Table S1: The summary of the fitted GEST with fixed coefficient of temperature and rainfall model for the national weekly cases of *Campylobacter*, the coefficient estimates of the model, the standard errors, the t values, and the significance level at 5%

Mu Coefficients	$\hat{\beta}_{1,j}$	std. error	t value	$Pr(>\|t\|)$
Intercept	6.640	1.985e-02	334.530	**2e-16**
trend	1.034e-03	8.316e-05	12.436	**2e-16**
Average temperature 2 weeks before	7.328e-03	1.303e-03	5.624	**6.24e-08**
Average total rainfall 1 week before	-2.439e-03	9.364e-04	-2.605	**0.00989**
Sigma Coefficients	$\hat{\beta}_{2,0}$	std. error	t value	$Pr(>\|t\|)$
Intercept	-4.7588	0.1019	-46.7	**2e-16**

Hence, the predictive mean number of cases of Campylobacter infection per week is:

$$\hat{\mu}_t = \exp\left[6.640 + 0.0073(temperature_{t-2}) - 0.0024(rainfall_{t-1}) + 0.0010(\zeta_t) + \hat{s}_{1,t}\right].$$

The coefficient of average temperature of two weeks before is positive and highly statistically significant, indicating an increase in the mean number of cases of Campylobacter due to the temperature of two weeks before. The coefficient of the total rainfall is negative and statistically significant, indicating a decrease in the mean number of cases of Campylobacter due to the total rainfall of one week before.

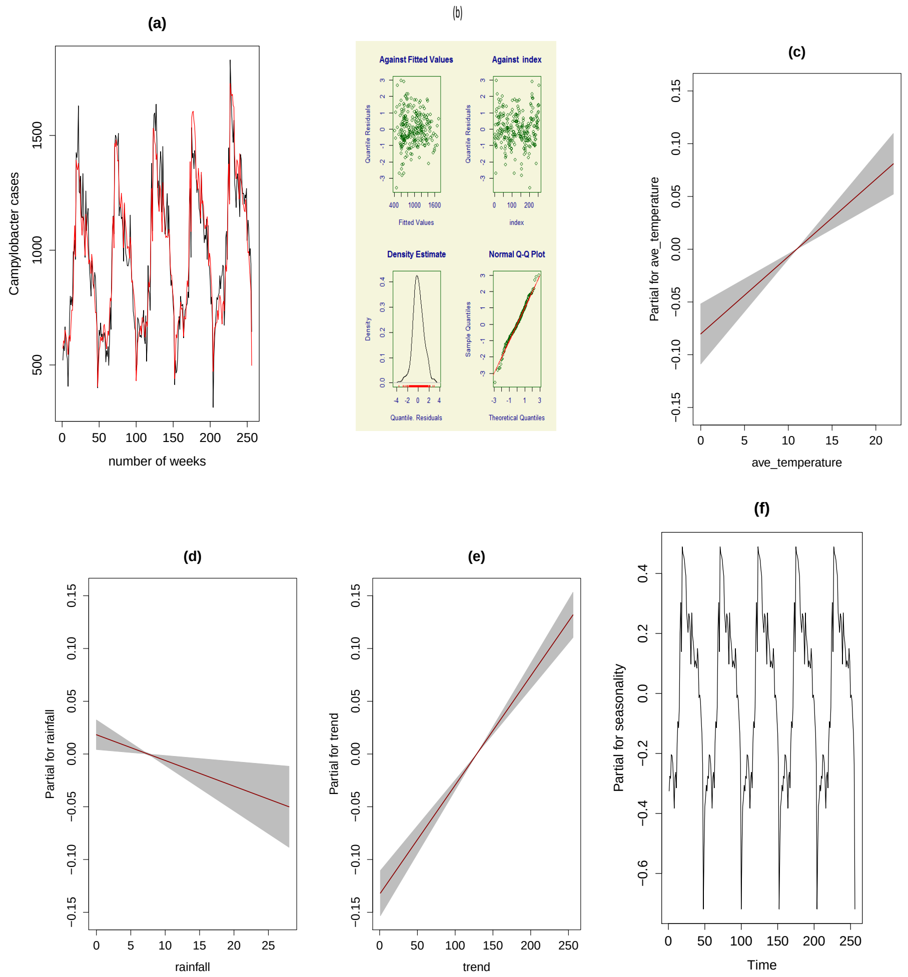

Figure S1: (a) Weekly number of cases of *Campylobacter* in England and Wales from February 2005 to December 2009 in black and the fitted mean in red; (b) QQ plot of the residuals, (c) estimated centered linear effect of average temperature, (d) of total rainfall, (e) and trend on the fitted mean number of cases of Campylobacter with 95% confidence interval, (f) estimated centered nonlinear effect of seasonality on the fitted mean number of cases of Campylobacter using GEST with fixed coefficient.

Table S2: The p values at 5% significance level of the coefficients of three fitted GEST models with different measures of temperature (average, maximum and minimum) of two weeks before diagnosis and total rainfall of one week before diagnosis with negative binomial distribution for Campylobacter cases in England and Wales, South East, East of England, South West, North West, North East, West Midlands, East Midlands, Wales, Yorkshire and the Humber, and London.

Trend and weather parameters	Nat	S.East	East	S.West	N.West	N.East	W.Mid.	E.Mid.	Wales	Y.H	London
Trend	.000	.000	.488	.000	.000	.000	.000	.000	.000	.000	.000
Ave temperature 2 weeks before	.000	.049	.000	.000	.180	.000	.000	.069	.964	.000	.375
Total rainfall 1 week before	.009	.563	.428	.085	.232	.396	.342	.483	.032	.009	.059
Trend	.000	.000	.358	.000	.000	.000	.000	.000	.000	.000	.000
Max temperature 2 weeks before	.000	.000	.000	.000	.000	.002	.000	.002	.222	.000	.188
Total rainfall 1 week before	.020	.599	.607	.103	.210	.334	.480	.535	.030	.028	.068
Trend	.000	.000	.544	.000	.000	.000	.000	.000	.000	.000	.000
Min temperature 2 weeks before	.000	.048	.000	.043	.017	.001	.000	.213	.777	.000	.005
Total rainfall 1 week before	.007	.753	.324	.085	.367	.429	0.23	.459	.030	.001	.038

Table S2 gives the p values at 5% significance level of the estimated parameters of the GEST with fixed coefficient of temperature and rainfall for national and regional weekly cases of Campylobacter in England and Wales. Three models of GEST were fitted with different measures of temperatures (average, maximum and minimum) of two weeks before. The table shows a strong statistical significance of the temperature in the prediction of Campylobacter cases in England and Wales, both at a regional and national level.

S2.2 GEST with varying coefficient of temperature

$$\begin{aligned} Y_t|\mu_t, \sigma_t &\sim \mathcal{NBI}(\mu_t, \sigma_t) \\ \log(\mu_t) &= \beta_{1,0} + f(temperature_{t-2}, weeks) + \beta_{1,2}(rainfall_{t-1}) + \beta_{1,3}(\zeta_t) + s_{1,t} \\ s_{1,t} &= -\sum_{m=1}^{M-1} s_{1,t-m} + w_{1,t} \\ \log(\sigma_t) &= \beta_{2,0} \end{aligned} \quad \text{(S3)}$$

where $f(temperature_{t-2}, weeks) = temperature_{t-2} * weeks + f(temperature_{t-2})$ is the interaction of a varying coefficient term, $weeks$, with the temperature plus a nonparametric function of the temperature, where $weeks$ is a factor with 13 levels, each level has four weeks period, $weeks = (1, 1, 1, 1, 2, 2, 2, 2, ..., 13, 13, 13, 13)$, and the nonlinear function f is modelled with P-spline, using "`pb`" and/or "`s`" routines from the packages `gamlss` and `mgcv` in R . $\zeta_t = 1, 2, \ldots, T$ is a fixed linear trend of time, $s_{1,t}$ is the time-varying seasonality with frequency $M = 52$ and a random error term $w_{1,t} \sim N_T\left(0, \sigma_w^2 \mathbf{I}_T\right)$.

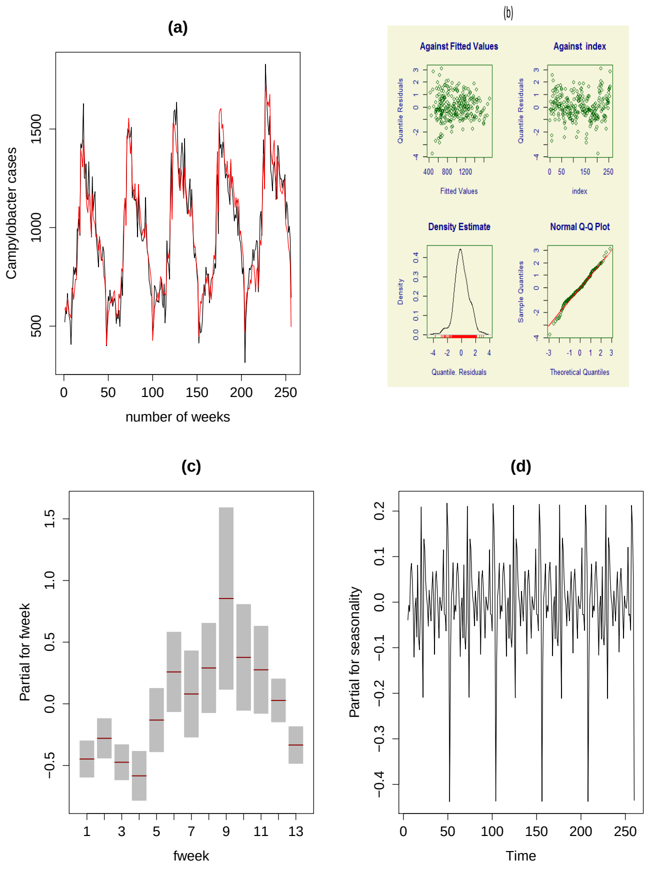

Figure S2: (a) Weekly number of cases of *Campylobacter* in England and Wales from February 2005 to December 2009 in black and the fitted mean in red; (b) QQ plot of the residuals, (c) estimated centered effect of coefficient term *weeks* on the fitted mean number of cases of Campylobacter with 95% confidence interval, (d) estimated centered nonlinear effect of seasonality on the fitted mean number of cases of Campylobacter, the big drops represent weeks 22, 35 and 52 of the calender year which corresponds to Easter, end of summer and Christmas bank holidays. The drop of the seasonality in these weeks could be due to minimum access to health care services.

S3 The contribution of temperature, rainfall, trend and seasonal terms to the burden of *Campylobacter*

The contribution of temperature, rainfall, trend and seasonal terms to the burden of *Campylobacter* was estimated by calculating the relative change in the predictive mean caused by each factor and averaging across the entire time-domain T. We compared between the GEST with fixed coefficient of temperature and rainfall model and the GEST with varying coefficient of temperature and fixed coefficient of rainfall. The purpose of this comparison is the see whether the interaction between temperature and weeks has an effect on the seasonality in the fitted mean number of Campylobacter cases.

In formulae:
Relative change in the mean number of cases due to the fixed effect of temperature:

$$\frac{1}{T}\sum_t \frac{\hat{\mu}_t - \hat{\mu}_t \mid_{No\ Temperature}}{\hat{\mu}_t} = \frac{1}{T}\sum_t \frac{\left[\exp\left(\hat{\beta}_{1,1} temperature_{t-2}\right) - 1\right]}{\exp\left(\hat{\beta}_{1,1} temperature_{t-2}\right)} \tag{S4}$$

where $\hat{\mu}_t$ is given by equation S2.

Relative change in the mean number of cases due to the varying effect of temperature:

$$\frac{1}{T}\sum_t \frac{\hat{\mu}_t - \hat{\mu}_t \mid_{No\ Temperature}}{\hat{\mu}_t} = \frac{1}{T}\sum_t \frac{\left[\exp\left(\hat{f}\left[temperature_{t-2}, weeks\right]\right) - 1\right]}{\exp\left(\hat{f}\left[temperature_{t-2}, weeks\right]\right)} \tag{S5}$$

where $\hat{\mu}_t$ is given by equation S3.

Relative change in the mean number of cases due to the effect of rainfall:

$$\frac{1}{T}\sum_t \frac{\hat{\mu}_t - \hat{\mu}_t \mid_{No\ Rainfall}}{\hat{\mu}_t} = \frac{1}{T}\sum_t \frac{\left[\exp\left(\hat{\beta}_{1,2} rainfall_{t-1}\right) - 1\right]}{\exp(\hat{\beta}_{1,2} rainfall_{t-1})} \tag{S6}$$

where $\hat{\mu}_t$ is given by equation S2, (S3).

Relative change in the mean number of cases due to the effect of trend:

$$\frac{1}{T}\sum_t \frac{\hat{\mu}_t - \hat{\mu}_t \mid_{No\ Trend}}{\hat{\mu}_t} = \frac{1}{T}\sum_t \frac{\left[\exp\left(\hat{\beta}_{1,3}\zeta_t\right) - 1\right]}{\exp\left(\hat{\beta}_{1,3}\zeta_t\right)} \tag{S7}$$

where $\hat{\mu}_t$ is given by equation S2, (S3).

Relative change in the mean number of cases due to the effect of seasonality:

$$\frac{1}{T}\sum_t \frac{\hat{\mu}_t - \hat{\mu}_t \mid_{No\ Seasonality}}{\hat{\mu}_t} = \frac{1}{T}\sum_t \frac{[\exp(\hat{s}_{1,t}) - 1]}{\exp(\hat{s}_{1,t})} \tag{S8}$$

where $\hat{\mu}_t$ is given by equation S2, (S3).

Table S3: Relative change in the mean number of *Campylobacter* cases due to the effect of trend, seasonality, temperature, and rainfall using the GEST with fixed coefficient analysis of temperature and rainfall

	trend%	seasonality%	temperature%	rainfall%
National	12.2	-3.4	7.7	-1.9
East	0.9	-3.1	13.3	-0.6
South West	7.9	-2.9	8.3	-1.9
North West	6.6	-4.8	3.5	-1.6
North East	12.2	-7.9	-17.6	-1.3
South East	23.7	-4.2	4.7	-0.6
West Midlands	14.6	-3.2	9.4	-0.9
East Midlands	19.3	-6.5	-5.7	1.0
Wales	9.8	-5.0	0.1	-3.0
York and Hum	7.4	-2.4	18.8	-3.1
London	13.4	-2.3	2.2	-2.1

A comparison of Table S3 with Table S4 shows that the relative change associated with the seasonality is much smaller in the model with varying coefficients, also the relative change associated with the temperature is much higher in the model with varying coefficients. This suggests that, in contrast with the model with fixed coefficients, the model with varying coefficients is able to capture most of the variability of *Campylobacter* cases (resulting in a smaller seasonal component), although understanding the underlying bio-physical mechanism requires further research.

Table S4: Relative change in the mean number of *Campylobacter* cases due to the effect of trend, seasonality, temperature, and rainfall using the GEST with varying coefficient analysis of temperature

	trend%	seasonality%	temperature%	rainfall%
National	12.0	-0.5	33.3	-1.7
East	0.8	-0.4	31.3	-0.4
South West	6.1	-0.6	23.6	-1.8
North West	6.4	-0.8	31.0	0.2
North East	13.1	-0.9	36.2	-0.7
South East	22.9	-0.8	36.4	-0.9
West Midlands	14.1	-0.7	29.1	-0.4
East Midlands	18.9	-1.1	26.7	2.0
Wales	9.5	-1.0	44.2	-2.9
York and Hum	7.3	-0.6	28.3	-2.5
London	13.1	-0.7	28.3	-2.8

S4 Hierarchical clustering analysis

Let Y_t be the weekly number of cases of *Campylobacter* infection in thirty three Strategic Health Authorities in England and Wales from January 1989 to December 2009, and consider the conditional negative binomial type I distribution $\mathcal{NBI}(\mu_t, \sigma_t)$ of the response variable Y_t in the GEST model, without explanatory variables and a fixed dispersion parameter. The fitted seasonality of the GEST for *Campylobacter* infections were clustered using the Ward's minimum variance algorithm in R.

$$\begin{aligned} Y_t|\mu_t, \sigma_t &\sim \mathcal{NBI}(\mu_t, \sigma_t) \\ \log(\mu_t) &= \beta_1 + \gamma_t + s_t \\ \gamma_t &= 2\gamma_{t-1} - \gamma_{t-2} + b_t \\ s_t &= -\sum_{m=1}^{M-1} s_{t-m} + w_t \\ \log(\sigma_t) &= \beta_2 \end{aligned} \tag{S9}$$

S4.1 Avon, Gloucestershire and Wiltshire SHA

Here is an example of GEST decomposition of *Campylobacter* cases in Avon, Gloucestershire and Wiltshire Strategic Health Authority with a conditional negative binomial distribution. The trend was fitted with a random walk order two and the seasonality was fitted with random seasonality of frequency 52 weeks.

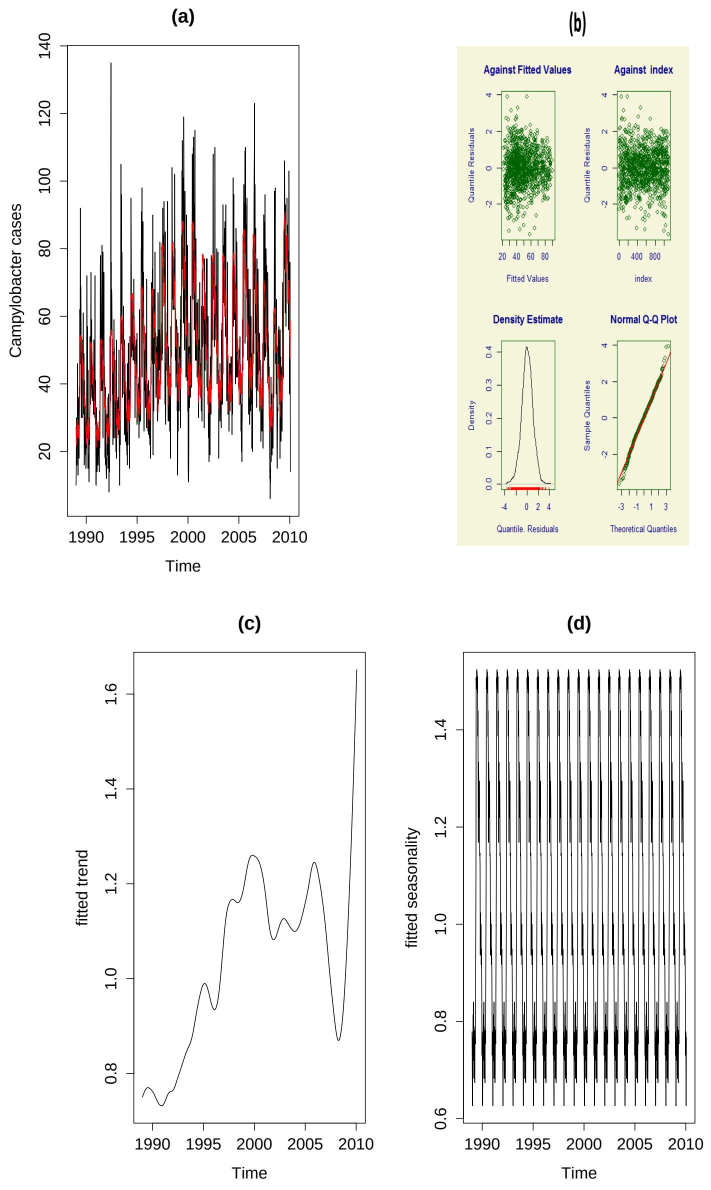

Figure S3: (a) Weekly number of cases of *Campylobacter* in Avon, Gloucestershire and Wiltshire Strategic Health Authority from January 1989 to December 2009 in black and the fitted mean in red; (b) QQ plot of the residuals, (c) the fitted trend, (d) the fitted seasonality.

S5 Wavelet analysis

Wavelet analysis decomposes the time series into a sum of wavelets, *i.e.* basic, time-localized, small, waves of a particular frequency (Cazelles 2007). Accordingly, the (continuous) wavelet transformation is the convolution:

$$W_x(a, \tau) = \frac{1}{\sqrt{a}} \int_{-\infty}^{\infty} x(t) \psi^* \left(\frac{t-}{a} \right) dt, \qquad \text{(S10)}$$

where $x(t)$ is the signal (*e.g.* the time series of *Campylobacter* cases) to be transformed, t is the time,$\psi(\frac{t-}{a})$ represents the wavelet (in general is a complex-valued function), the subscripts x refer to the time series under study, and the superscript '$*$ denote the complex conjugate form. Here we used a well-established functional forms for the wavelet (the Morlet wavelet) for which the scale a is almost equal to the period of the wavelet (Torrence 1998). The squared wavelet transform, $W_x(a, \tau)W_x^*(a, \tau)$ is referred as power spectrum which is usually displayed as a contour plot on a time-period plane. The power spectrum quantifies the contribution of the various components of the time series x(t) of different periods (also denoted as 'harmonics) at different times (Cazelles 2007). The power spectrum averaged over the entire time domain is referred to as 'global power spectrum.

Wavelet analysis can be applied to compare two different non-stationary time series, e.g. disease incidence and weather time series. This is usually done by studying the wavelet cross-spectrum and wavelet coherence. The cross-spectrum is defined as the product of the single wavelet transforms the two different time series x(t) and y(t) i.e. $W_x(a,)W_y^*(a,)$; the wavelet coherency is defined as the cross-spectrum normalized by the spectrum of each time series after smoothing in both time and scale. The wavelet coherency, which is bounded between 0 and 1, quantifies the strength of a linear relationship between the various corresponding harmonics of two non-stationary time series at different times. Finally, possible time-lags between the harmonics of the two time series can be detected by studying the phase difference which can be obtained by appropriate relationship between the imaginary and the real part of the wavelet cross-spectrum (Cazelles 2007).

The analysis of reported *Campylobacter* infections was strongly affected by under-reporting during weekend and Bank Holidays (the reported cases per day of the week consistently dropped on Saturdays and Sundays). The wavelet analysis of the power spectrum and the global spectrum exhibited a strong weekly seasonality, with an additional 3.5 day harmonic (Figure S5). The strong peaks at 3.5 and 7 days were removed by using adjusted data with a seven day rolling mean to correct for day of week and bank holidays (Nichols 2012)

Figure S5 shows: a) Average weekly reported *Campylobacter* cases averaged over 20 years (from 1989 to 2009). All time series were square root transformed and then normalised to sum to unity. c)wavelet power spectrum of the transformed time-series of *Campylobacter*. Low values of the power spectrum are shown in dark blue, and high values in dark red. The dotted white lines

show the maxima of the undulations of the wavelet power spectrum and the dotted-dashed black lines show the 5% significant levels computed based on 100 bootstrapped series. The light blue shaded areas identify the region subjected to errors arising from dealing with a finite-length time series (edge effect). e) global average wavelet power spectrum g) original and reconstructed time-series according to all harmonics and the selected first 3 harmonics only. b), d), f) h) As in figures a),c) e) and g) but after the time-series of *Campylobacter* cases were adjusted using a seven day rolling mean, removal of bank holiday artefacts and adjusted for long term trend.

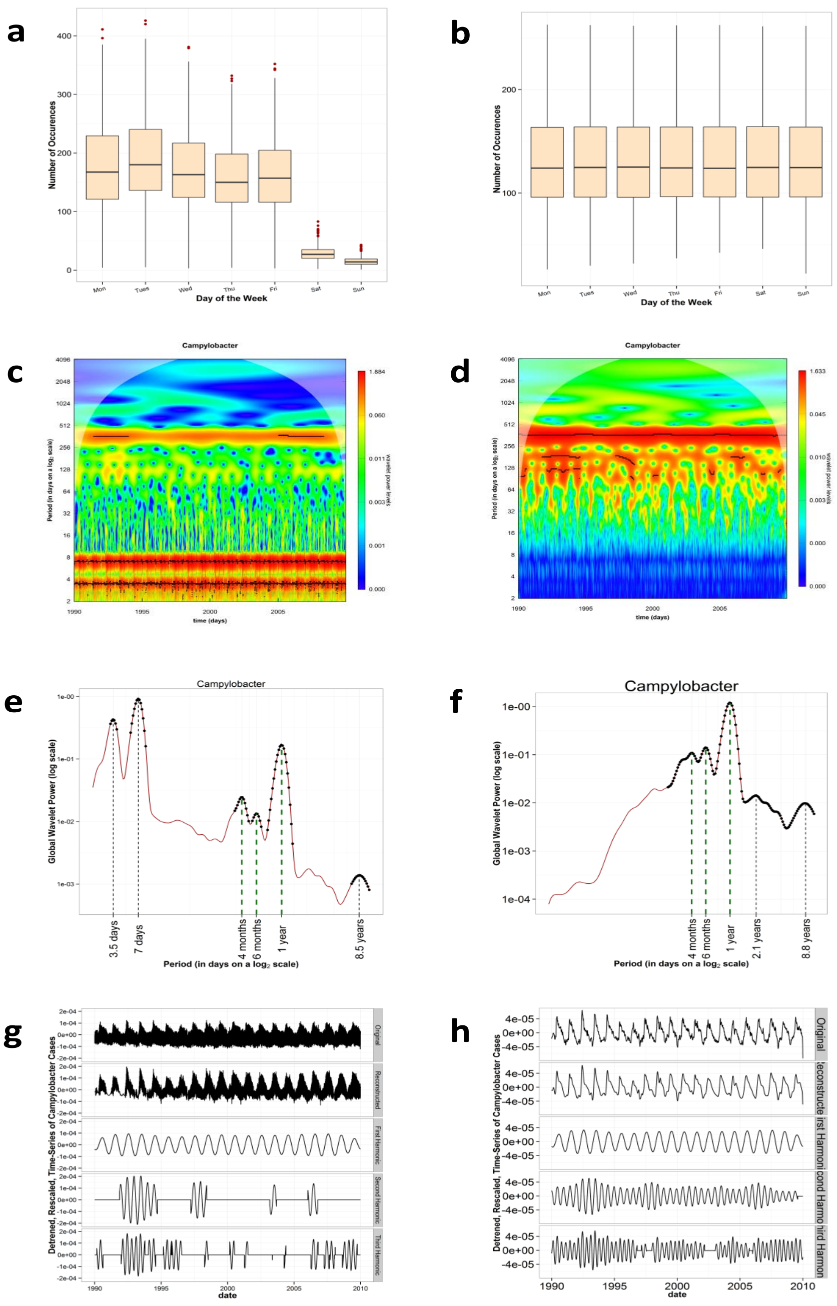
a
b
c
d
e
f
g
h
Number of Occurences
Day of the Week
Mon
Tues
Wed
Thu
Fri
Sat
Sun
Campylobacter
Period (in days on a log2 scale)
time (days)
wavelet power levels
Global Wavelet Power (log scale)
3.5 days
7 days
4 months
6 months
1 year
8.5 years
2.1 years
8.8 years
Detrened, Rescaled, Time-Series of Campylobacter Cases
Original
Reconstructed
First Harmonic
Second Harmonic
Third Harmonic
date
